# Chicago Academy of Natural Sciences

**Published:** 1857-03

**Authors:** 


*Chicago Academy of Natural Sciences.*

As members of the medical profession are everywhere the most zealous cultivators of the natural sciences, we do not hesitate to copy the following from one of the daily papers of this city. We trust the Society will soon have in Chicago a Museum that will be an honor to the city, and an object of interest to all naturalists.

'*The Chicago Academy of Natural Sciences.*—At a recent meeting of gentlemen, interested in scientific pursuits, a Society was organized under the above title. A constitution was adopted and the following officers were elected:—

'*President*—Prof. J. V. Z. Blaney.

'*Vice-Presidents*—Prof. N. S. Davis, Capt. J. D. Webster.

'*Corresponding Secretary*—Prof. H. A. Johnson.

'*Recording Secretary*—Dr. Henry Parker.

'*Treasurer*—Col. R. K. Swift.

'*Curator of Museum and Library*—Prof. E. Andrews.

'The following gentlemen were appointed chairmen of standing committees on the various branches of natural sciences:—

'Zoology, Robert Kennicott; Botany, Dr. F. Scammon; Geology, J. A. Lapham; Chemistry, Prof. Blaney; Microscopy and Physics, H. Zimmerman.

'The first means proposed by the Society for the promotion of its objects, is the formation of a museum of scientific specimens and a library. A room for this purpose has been procured in the Dearborn Seminary building, and a collection of several thousand specimens already awaits the construction of cases to receive them. Other extensive and valuable contributions are promised. With the requisite aid from our public-spirited citizens to defray the necessary expenses, such as procuring cases, and providing for the proper care and preservation of specimens, a good beginning can be made at once.

'The objects of the Society commend themselves to all friends of science and education. The natural sciences would seem particularly fitted to interest and profit our Western people. These sciences seek to interpret nature in her grandest form, while several of them, as chemistry, geology, and mechanics, relate directly to our economical interests, and lie at the foundation of the industrial civilization of our times.

'All friends of the various educational institutions growing up in our city, should lend a helping hand to this Society. Their students will need access to a cabinet for the study of mineralogy, geology, and zoology. And will it not be better to unite their efforts, and make one extensive and valuable museum, than for each to attempt to have one of its own, and so have none of any pretensions to completeness? It is proposed to give students of these institutions access to the collections of the Society, for the purpose of lectures and inspection. The cabinet will also be open to all interested in the examination of the subjects to which it will relate.'

